# Snowball Group Usability Testing for Rapid and Iterative Multisite Tool Development: Method Development Study

**DOI:** 10.2196/55316

**Published:** 2025-02-18

**Authors:** Katherine L Dauber-Decker, David Feldstein, Rachel Hess, Devin Mann, Eun Ji Kim, Pranisha Gautam-Goyal, Jeffrey Solomon, Sundas Khan, Fatima Malik, Lynn Xu, Ainsley Huffman, Paul D Smith, Wendy Halm, Alice Yuroff, Safiya Richardson

**Affiliations:** 1 Feinstein Institutes for Medical Research, Northwell Health, 600 Community Drive, Suite 403, Manhasset, NY, 11030, United States, 1 5166001421; 2 Northwell Health, 2000 Marcus Avenue, Suite 300, New Hyde Park, NY, 11042, United States, 1 5166001421; 3 University of Wisconsin School of Medicine and Public Health, Madison, WI, United States; 4 University of Utah Health, Salt Lake City, UT, United States; 5 Department of Population Health, NYU Grossman School of Medicine, New York, NY, United States; 6 Department of Health Informatics, Medical Center Information Technology, NYU Langone Health, New York, NY, United States; 7 Donald and Barbara Zucker School of Medicine at Hofstra/Northwell, Manhasset, NY, United States; 8 Department of Medicine, Baylor College of Medicine, Houston, TX, United States; 9 Center for Innovations in Quality, Effectiveness and Safety, Michael E. DeBakey VA Medical Center, Houston, TX, United States; 10 Utah Clinical and Translational Science Institute, University of Utah, Salt Lake City, UT, United States; 11 University of Wisconsin School of Nursing, Madison, WI, United States; 12 Institute for Excellence in Health Equity, NYU Langone Health, New York, NY, United States

**Keywords:** clinical decision support, CDS, decision aid, clinical aid, cough, sore throat, strep pharyngitis, snowball group usability testing, snowball group, usability testing

## Abstract

**Background:**

Usability testing is valuable for assessing a new tool or system’s usefulness and ease-of-use. Several established methods of usability testing exist, including think-aloud testing. Although usability testing has been shown to be crucial for successful clinical decision support (CDS) tool development, it is often difficult to conduct across multisite development projects due to its time- and labor-intensiveness, cost, and the skills required to conduct the testing.

**Objective:**

Our objective was to develop a new method of usability testing that would enable efficient acquisition and dissemination of results among multiple sites. We sought to address the existing barriers to successfully completing usability testing during CDS tool development.

**Methods:**

We combined individual think-aloud testing and focus groups into one session and performed sessions serially across 4 sites (snowball group usability testing) to assess the usability of two CDS tools designed for use by nurses in primary and urgent care settings. We recorded each session and took notes in a standardized format. Each site shared feedback from their individual sessions with the other sites in the study so that they could incorporate that feedback into their tools prior to their own testing sessions.

**Results:**

The group testing and snowballing components of our new usability testing method proved to be highly beneficial. We identified 3 main benefits of snowball group usability testing. First, by interviewing several participants in a single session rather than individuals over the course of weeks, each site was able to quickly obtain their usability feedback. Second, combining the individualized think-aloud component with a focus group component in the same session helped study teams to more easily notice similarities in feedback among participants and to discuss and act upon suggestions efficiently. Third, conducting usability testing in series across sites allowed study teams to incorporate feedback based on previous sites’ sessions prior to conducting their own testing.

**Conclusions:**

Snowball group usability testing provides an efficient method of obtaining multisite feedback on newly developed tools and systems, while addressing barriers typically associated with traditional usability testing methods. This method can be applied to test a wide variety of tools, including CDS tools, prior to launch so that they can be efficiently optimized.

## Introduction

Usability testing assesses a new tool or system’s usefulness and ease-of-use. Usability testing is particularly important in clinical decision support (CDS) development. CDS tools are evidence-based tools that help clinicians make decisions to improve patient care. Usability testing during CDS development increases the likelihood of tool adoption and impact by creating a tool that is workflow-integrated, useful, and easy to use [1-5]. The current standard for usability testing of CDS tools is think-aloud testing in which approximately 3‐5 end users are asked to think out loud while interacting with a prototype of the tool during individual sessions.

Although the benefits of usability testing are well established, it is often not conducted during typical CDS development due to tight project timelines. Think-aloud testing with 3‐5 users may add weeks to a project timeline. This becomes a larger challenge in multisite development projects. Typically, usability testing is done on the same tool at multiple sites in parallel, where end-users at each site are likely to report similar issues, adding weeks to the project timeline at each site with minimal added value of testing at each site. An efficient, effective method for usability testing CDS across multiple sites is needed.

In addition to think-aloud testing done in individual sessions, focus groups are also used to obtain feedback on a tool. These two types of testing can provide different types of insight from future end-users [6,7]. Focus groups include interactions between different participants, which can lead to the development of consolidated ideas through conversation. However, a single vocal participant can influence the direction of the discussion and may not represent all the participants’ views. One-on-one interviews, in contrast, enable the research team to discuss individual-level feedback with each participant [7]. Including both types of usability testing allows for the highest yield of testing [1,4].

We sought to develop a new method of usability testing, termed snowball group usability testing, that would enable efficient, high-yield multisite testing. We applied our method to a project aimed at decreasing antibiotic overprescribing in the outpatient setting. In the United States, antibiotics are frequently prescribed inappropriately, contributing to antibiotic resistance [8-13]. The goals of our testing were to (1) determine the feasibility and practicality of snowball group usability testing and (2) facilitate the development of two useful and usable CDS tools. Here we describe our method for snowball group usability testing.

## Methods

### Ethical Considerations

All study activities were approved by the New York University Langone Institutional Review Board (i19-01222). Verbal informed consent was obtained from all usability testing participants. Any identifiable data collected as part of the study were stored on secure drives and only accessible to members of the study team. Participants were not compensated for partaking in this research study. This manuscript does not include any identifiable study data.

### Setting and Recruitment

We conducted this study at 4 large academic sites in New York, Wisconsin, and Utah. The study teams at each site were experienced in CDS design, development, and evaluation, with a focus on using human-centered design strategies. Nurses who were eligible to participate in usability testing were identified based on their roles at study clinics and eligibility criteria. The study was presented to nurses by the study team. Eligible nurses were contacted by email for recruitment. Nurses who were interested in participating provided verbal consent and were given a key information sheet that outlined their research participation.

### Description of CDS Tools

**Figure 1.**
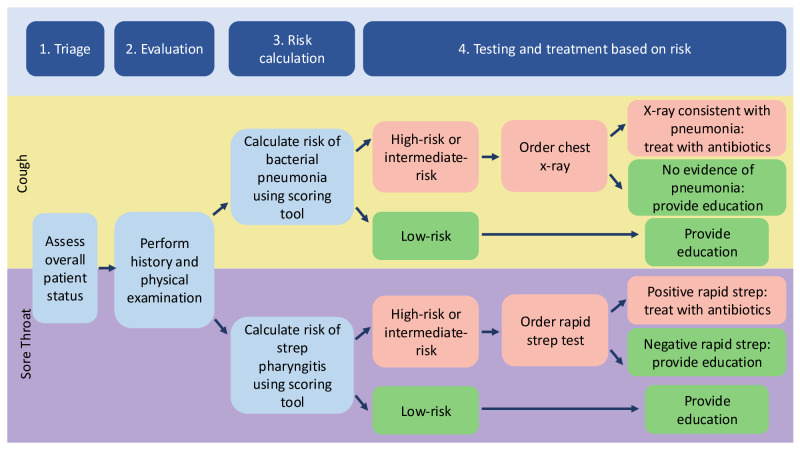
Cough and sore throat clinical decision support workflow diagram. The workflow for both tools included 4 steps: (1) triage, (2) evaluation, (3) risk calculation, and (4) testing and treatment based on risk.

For this study, we used the Heckerling rule for cough [14] and the Centor score for sore throat [15-17] to develop CDS tools for use by nurses in an outpatient setting. We developed each of these tools for use in two different electronic health records (EHRs), Epic and Allscripts TouchWorks, due to site differences. Each tool included an in-person nurse visit section and a triage section that could be applied either over the phone or in-person. During triage, the nurse or medical assistant recorded the patient’s symptoms using a tool that helped determine the risk and severity level for the patient. This was used to determine if the patient needed to be seen in person for those who were assessed over the phone. Patients who needed to be seen or were already in the office but did not have severe symptoms or other significant illnesses were deemed eligible for an in-person nurse visit. Alternatively, the patient could be sent to an emergency department or physician visit, or for those who were assessed over the phone, they could be advised that they do not need a visit and could self-treat with supportive care at home (Figure 1). The assessment questions were developed based on current site standards for patients with cough and sore throat [18].

If a nurse visit was deemed appropriate for the patient, the nurse completed the visit section during an in-person encounter. The visit tools included a note for documenting history and the physical exam, a risk calculator to determine the risk of bacterial infection, and order sets based on the results of the risk calculator. Orders could be placed or pended by nurses to be signed by providers. The risk calculators were based on the Heckerling rule or Centor score for cough or sore throat, respectively, and recommendations were given based on the overall score (Table 1). Based on the number of criteria with which the patient presented, rule users could stratify patients into low-, intermediate-, or high-risk categories, indicating different levels of care.

**Table 1.** Heckerling rule and Centor score criteria.

Rule	Criteria	Low-risk	Intermediate- or high-risk
Heckerling rule (pneumonia)	Temperature of >100°F (37.8 °C) Heart rate of >100 beats/min Crackles (rales) Decreased breath sounds No history of asthma	0‐1: supportive care indicated	2‐5: chest x-ray and antibiotics if x-ray was positive
Centor score (strep pharyngitis)	History of fever: feels feverish, sudden onset of cold with shivering sweats, or oral temperature of ≥100.4°F (38.0 °C) Absence of cough: new onset, frequent and/or persistent, different from baseline Tonsillar exudates Tender anterior cervical nodes	0‐1: no rapid strep test indicated	2‐3: rapid strep test indicated and antibiotics if the test is positive

### Snowball Group Usability Testing Sessions

We combined individual think-aloud sessions and focus groups into one session. We conducted these sessions at each site serially, with the hope that each subsequent organization would reach saturation more quickly by benefitting from the previous site’s insights. This form of testing will hereafter be referred to as snowball group usability testing, in which “snowballing” refers to conducting usability testing serially at each site, transferring feedback regarding the CDS tools from site to site as testing progresses, and “group” refers to conducting testing with multiple participants at once. To our knowledge, snowballing has not previously been used in the context of usability testing. We applied snowballing with the goal of increasing the efficiency at which results were obtained and passed along between study institutions. Each site performed a minimum of one group think-aloud session for the cough tool and another for the sore throat tool with one exception. Site 4 did not complete a cough CDS testing session. Sessions were conducted with the EHR encoded tool within a sandbox environment (for the Epic EHR sites) or a Research Electronic Data Capture (REDCap) mock-up of the tool (for the Allscripts TouchWorks EHR site). Each session was structured to include an individual think-aloud component with 1 to 4 nurse participants (and in one session, a medical assistant) simultaneously, followed by a focus group component with all participants that included debriefing questions about the tool. Results were documented in a pretemplated format for rapid dissemination to the other study sites to enable the efficient integration of feedback prior to the next site’s testing session. Sessions were completed remotely on Microsoft Teams, Cisco Webex, or Zoom depending on site requirements.

Study team moderators were different for each site due to Institutional Review Board regulations (ie, study team members at each site could only conduct testing with their own site’s participants). The main moderator presented an overview of the session and the tools. This included instructions on how to moderate think-aloud sessions. To obtain individualized feedback, each nurse worked with an additional session moderator in an individual web-based breakout room. Here, the nurse used the think-aloud method while navigating through the tool or a prototype of the tool. Each nurse used the tool or prototype on mock clinical cases. The main moderator circulated among the breakout rooms throughout the session to answer any questions, resolve any issues, and ensure that the session ran smoothly.

Following the individual breakout room think-aloud sessions, all nurse participants were brought back together into the main meeting room for a focus group to answer debriefing questions about their opinions of the tool. All participants were encouraged to speak, and individual participants were asked for their thoughts if they were not frequently volunteering their opinions. Sites shared their results with each other between sessions so that any necessary tool modifications could be made prior to the next testing session, enabling rapid iteration of the tools. Sites requiring more feedback following their initial sessions completed an additional session.

### Snowballing Approach to Method Development

Prior to initiating our usability testing sessions, we assembled a reporting form template in PowerPoint that we could quickly fill in with structured feedback from our sessions. As each site ran their testing session, and moderators filled out the template with session results and shared it with the other participating sites. This enabled the other sites to make changes as needed before conducting their own usability testing sessions. In addition, the PowerPoint template included space for sites to share information about how to iteratively adjust the methods to efficiently run the sessions based on their experiences. Each site highlighted any challenges they had with completing the sessions and suggestions for how to avoid such challenges in future sessions, allowing sites to implement changes as needed. With the goal of rapidly iterating on our tools among sites, we did not use traditional in-depth thematic analysis in favor of quick feedback summaries. Our informal thematic analysis enabled us to quickly resolve the most easily addressable issues between sessions.

## Results

A summary of our session durations and participant breakdowns is provided in Table 2.

**Table 2.** Time and participant breakdown of snowball group usability testing sessions.

Variable	Average value, mean	Site 1	Site 2		Site 3	Site 4^a^
		Session 1	Session 1	Session 2^b^	Session 1	Session 1
Cough clinical decision support testing						
Participants, n	3.3	3	4	—	3	—
Facilitators, n	4.7	4	4	—	6	—
Think-aloud time (min)	35.8	30	30‐45^c^	—	40	—
Focus group time (min)	18	21	—^d^	—	15	—
Sore throat clinical decision support testing						
Participants, n	3	4	2	1	3	5
Facilitators, n	4	4	4	3	6	3
Think-aloud time (min)	36.4	27	40	45	30	40
Focus group time (min)	14.1	24	—^d^	5	17.5	10

a Site 4 did not complete usability testing sessions for the cough tool.

b Site 2 completed 2 sessions to test the sore throat tool.

c This range represents differences in the amount of time participants were able to take part in the session.

d This session did not include a focus group component.

### Cough and Sore Throat CDS Tool Feedback

**Figure 2.**
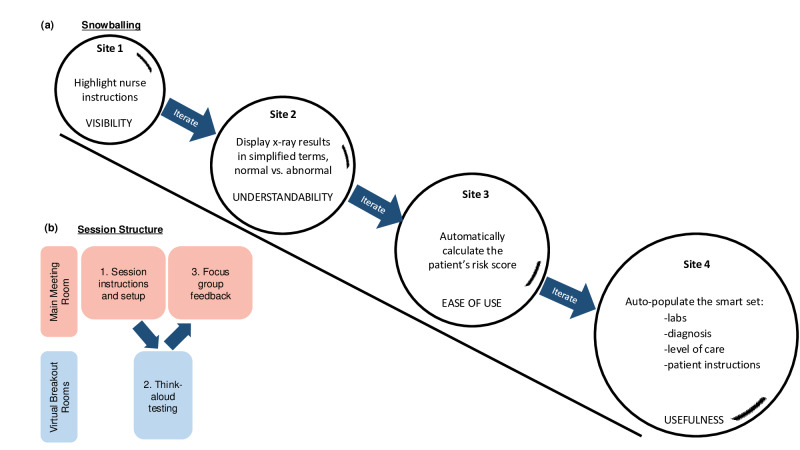
Snowball group usability testing enabled efficient acquisition of iterative feedback across study sites. (**A**) Snowballing enabled iterative integration of feedback between testing sessions. Circles indicate feedback obtained at during each site’s testing sessions. Snowballing is indicated by the increasing size of the circles from site to site. (**B**) Each session included both think-aloud testing and focus group components except where indicated in Table 2.

Snowball group usability testing enabled us to efficiently obtain and incorporate iterative feedback across our study sites (Figure 2). Overall, participants felt that the CDS tools were useful but identified some areas for potential improvement. Overarching themes from the sessions included suggestions for changes in the tool’s wording and formatting, as well as changes to the overall workflow. For example, participants identified the wording of “recent travel” as unclear, as they were unsure what timeframe would constitute recent. Similarly, “disease exposure” contained ambiguity as to which diseases the exposure could encompass. In terms of workflow, participants at one site pointed out that it would not make sense for the tool to ask about COVID results during the visit, as this would have been a prerequisite to the patient coming in for a visit at the time this study was conducted. Each of these suggestions provided valuable feedback for modifications to be made prior to the next testing session.

### Focus Group Testing Component

The focus group testing component of our new method proved to be highly beneficial. We asked debriefing questions regarding participants’ opinions of the tool in a focus group setting. One key feature was that, by collecting feedback from multiple participants at once rather than over a series of multiple separate sessions, we were more easily able to notice similarities in feedback among participants. In addition, each site’s study team was able to meet soon after the single group session, rather than once after several sessions had been completed. This made it easier to discuss results, find similarities in participant feedback, and generalize, due to the recency of the information being obtained.

### Snowballing Component

The snowballing component of our method also proved to be beneficial. A key benefit of snowballing was that study teams were able to incorporate changes based on previous sites’ sessions. For example, nurses at one site did not properly follow instructions for the order of how to choose antibiotics based on patient allergies. The study team made changes to the cough and sore throat CDS tools at this site to highlight these instructions. These changes were also incorporated into the CDS tools under development at the 2 other sites that used Epic EHRs in the study. In addition, during the first site’s testing session, it was determined that the location of the patient comorbidities, used to determine if a nurse visit was appropriate during triage, was not optimal. Comorbidity information was moved to below the nurse visit symptoms to improve ease of use. This feedback was incorporated into the second site’s tool prior to their usability testing session.

While snowballing was useful for identifying and implementing certain changes to the CDS tools among sites, differences in the 2 EHRs and their respective limitations precluded some tool components from being transferable between sites. For example, while the Epic EHR versions of the tools were able to automatically calculate results, the TouchWorks EHR versions lacked this capability due to system limitations. In addition, a key feature of the Epic CDS tool was that it could link to order sets, enabling the user to seamlessly proceed from the CDS to the appropriate orders for a patient. TouchWorks did not support connection to order sets, and therefore, the transition from the CDS to ordering in the TouchWorks versions of the CDS tools was more manual. This, unfortunately, could not be changed. Finally, differences in primary care and urgent care workflows precluded uniformity in our approaches in these two environments.

## Discussion

### Principal Findings

Snowball group usability testing is a novel method that can be used to rapidly and iteratively test the usability of a new CDS tool or workflow. To our knowledge, this is the first time usability testing has been performed in this manner. We identified 3 main benefits of snowball group usability testing. First, by interviewing several participants in a single session rather than individuals over the course of weeks, each site was able to rapidly obtain a large amount of data at once. With traditional testing approaches, in which one participant is interviewed at a time and each must be scheduled separately, it may take weeks or even months to obtain the same amount of data. Importantly, snowball group usability testing helped to expedite the overall usability testing timeline. By using our method sequentially (or in limited cases, concurrently) and at different sites, we were able to rapidly make changes, then retest our CDS tools with the next group of participants.

Second, combining the individualized think-aloud component with a focus group component in the same session helped us obtain a variety of feedback. In a focus group, one participant’s feedback can become amplified, as a more talkative participant may dominate the conversations, and others may simply agree with their thoughts. Similar to any other focus group, session moderators encouraged all nurses in the group to contribute to the conversation, calling out individuals if they had not provided feedback after a while. By combining methodologies, we first obtained in-depth individual-level feedback with the think-aloud sessions and then elicited group-based feedback with the focus groups when participants built upon each other’s opinions. Both types of feedback were useful; in the think-aloud sessions, the study team mostly observed, whereas in the focus group, the study team was more involved in asking for feedback on how to improve the tool. Therefore, combining approaches enabled us to get different types of feedback from the same set of participants all at once, which was invaluable when considering tight research and development timelines.

Third, our method enabled us to identify new and varied feedback from each site because we were able to edit key issues with our tools between sessions. Rather than hearing the same feedback in each session and making all of the changes at the end, our method resulted in a greater variety of feedback and a greater ability to optimize the tools.

Expediting the timeline and maximizing the amount of feedback gained are particularly valuable when developing EHR tools due to the inherent time constraints in the development process. Working with multiple sites and internal teams, such as those in charge of coding and implementing changes to the EHR, poses its own timing challenges as a result of the need to coordinate schedules and workloads. Thus, usability testing is often not considered a top priority; however, it remains an essential component of tool and system development. The use of snowball group usability testing effectively addresses this problem by reducing the time it takes to obtain and share feedback among participating sites. We also performed our testing remotely, which further addressed the issue of tight timelines and schedules, as participants did not have to travel from their clinics to be in the same location as other participants and the study team for the testing sessions. This enabled participants from different clinics to participate at the same time, without taking extra time out of their busy schedules for travel. Of note, remote testing can be used to mitigate scheduling issues with other forms of usability testing, including single-participant think-aloud sessions, interviews, and focus groups.

### Limitations

There were challenges and limitations with snowball group usability testing. We experienced some incompatibility between different EHR systems, limiting transferability. Although the content of the CDS tools was transferable, the overall structure of the note and workflow were quite different. In addition, there were some components of the tools (such as the automatic calculator and linking to order sets) that were possible in one system but not the other, requiring site-specific modifications and testing.

In addition, timing, staffing, and availability issues presented a challenge. There were variations in each site’s experience with usability testing. Finding 4‐5 experienced usability testing moderators for a given session was challenging for some sites. As a result, staff needed to be trained in the methodology, but identifying times when staff were available was challenging. Another site experienced research staff turnover at the beginning of the usability testing phase of our study, leading to a need for extra training and reshuffling of resources. Additionally, nurses participating in the sessions had limited time available to complete their sessions and the study teams were unable to get through all of their planned cases and questions. For example, in one session, only think-aloud testing was completed due to time constraints, as the nurses had competing clinical demands. In the same session, one of the nurse participants was frequently interrupted by medical staff and phone calls. Two sites cut the think-aloud session short, setting a time limit to ensure that the focus group component was included. Additional challenges with time included accounting for the different speeds at which participants gave feedback and a lack of opportunity for the study team to iteratively improve the timing issues from session to session, as most sites only conducted one. However, learning from earlier sessions, we were able to change future sessions so that each nurse started off with a different mock case during their respective think-aloud sessions. This ensured that each case was tested, even though each nurse did not complete every case themselves.

Finally, an additional limitation was the fact that, due to the pragmatic nature of our study, we did not perform highly rigorous qualitative or quantitative evaluations of our usability testing results. Future testing of this method should include validated usability measures, such as the System Usability Scale [19], in all rounds of testing, as well as qualitative thematic analysis to rigorously assess the impact of snowball group usability testing on the usability of the CDS tools being developed. However, based on our collective years of experience performing usability testing, the suggestions and improvements derived of from snowball group usability testing were on par with what we would expect to obtain from established usability testing techniques, and the quality of our findings was higher than what we have seen using previous testing methods.

### Conclusion

Snowball group usability testing provides a novel, efficient method of obtaining feedback on newly developed tools and systems, while addressing barriers typically associated with traditional usability testing methods. We successfully developed and used this method to test two CDS tools and rapidly iterated the process among 4 sites. Snowball group usability testing can be applied to test a wide variety of tools and workflows, including CDS tools, prior to launch so that they can be efficiently optimized, ultimately leading to higher adoption and end-user satisfaction.

## Abbreviations

CDS: clinical decision support
EHR: electronic health record
REDCap: Research Electronic Data Capture
